# High-precision regressors for particle physics

**DOI:** 10.1038/s41598-024-52941-4

**Published:** 2024-03-04

**Authors:** Fady Bishara, Ayan Paul, Jennifer Dy

**Affiliations:** 1 Deutsches Elektronen-Synchrotron DESY, Notkestraße 85, 22607 Hamburg, Germany; 2 Electrical and Computer Engineering, Northeastern University, 360 Huntington Ave., Boston, MA 02115 USA; 3 The Institute of Experiential AI, Northeastern University, 360 Huntington Ave., Boston, MA USA

**Keywords:** Experimental particle physics, Phenomenology, Theoretical particle physics, Scientific data, Computational science, Statistics

## Abstract

Monte Carlo simulations of physics processes at particle colliders like the Large Hadron Collider at CERN take up a major fraction of the computational budget. For some simulations, a single data point takes seconds, minutes, or even hours to compute from first principles. Since the necessary number of data points per simulation is on the order of $10^9$–$10^{12}$, machine learning regressors can be used in place of physics simulators to significantly reduce this computational burden. However, this task requires high-precision regressors that can deliver data with relative errors of less than 1% or even 0.1% over the entire domain of the function. In this paper, we develop optimal training strategies and tune various machine learning regressors to satisfy the high-precision requirement. We leverage symmetry arguments from particle physics to optimize the performance of the regressors. Inspired by ResNets, we design a Deep Neural Network with skip connections that outperform fully connected Deep Neural Networks. We find that at lower dimensions, boosted decision trees far outperform neural networks while at higher dimensions neural networks perform significantly better. We show that these regressors can speed up simulations by a factor of $10^3$–$10^6$ over the first-principles computations currently used in Monte Carlo simulations. Additionally, using symmetry arguments derived from particle physics, we reduce the number of regressors necessary for each simulation by an order of magnitude. Our work can significantly reduce the training and storage burden of Monte Carlo simulations at current and future collider experiments.

## Introduction

Particle physics experiments like those at the Large Hadron Collider at CERN^1–3^, are running at progressively higher energies and are collecting more data than ever before ^4^. As a result of the increase in the volume of data collected as well as improved analysis techniques and detector upgrades, the experimental precision of the measurements they perform is continuously improving. However, to infer what these measurements mean for the interactions between the fundamental constituents of matter, they have to be compared with, and interpreted in light of, our current theoretical understanding. This is done by performing first-principles computations for these high energy processes order by order in a power series expansion. After the computation, the resulting function is used in Monte Carlo simulations. The successive terms in the power series expansion, simplistically, become progressively smaller. Schematically, this can be written as:

1 $$\begin{aligned} F( \user2{x} ) = f_{00}( \user2{x} ) + \alpha \,f_{01}( \user2{x} )+ \alpha ^2\,\left\{ f_{11}( \user2{x} ) + f_{02}( \user2{x} )\right\} + \cdots \end{aligned}$$

where $\alpha \ll 1$ is the small expansion parameter. The functions, $f_{ij}$ are proportional to the real part of a complex number, $f_{ij}\propto \Re \{\textsf{M}^*_i\textsf{M}_j\}$, and $i+j$ is equal to the power of the expansion coefficient, that is a generic term is of the form $\alpha ^{i+j}\,f_{ij}$. Here, $\textsf{M}_i\in \mathbb {C}$ with $i\in \{0,1,2,\ldots \}$ is called the matrix element. For a given scattering process, it is computed by summing over all the contributing Feynman graphs and the subscript corresponds to the number of closed loops in those graphs. The term of interest to our current work is the one enclosed by the curly braces in Eq. (1) which we will refer to as the second-order term (Note: here *order* refers to the power of the expansion coefficient $\alpha$). The function, $F( \user2{x} )$, must be evaluated on the order of $10^9$–$10^{12}$ times for each simulation. However, for many processes, evaluating the second-order term, specifically, $f_{02}$, is computationally space- and time-intensive and could take several seconds to compute a single data point. It is much slower to evaluate than $f_{11}$ which appears at the same order because $f_{02}$ involves Feynman graphs with two closed loops which are much more complicated than the one-loop ones that enter the $f_{11}$ term. Moreover, these samples cannot be reused leading to an overall high cost of computation for the entire process under consideration. Building surrogate models to speed up Monte Carlo simulations is highly relevant not only in particle physics but in a very large set of problems addressed by all branches of physics using perturbative expansion like the one in Eq. (1). We give a broader overview of the physics motivation and applications in “Physics context”. The bottleneck we address in this work is only a part of the full Monte Carlo simulation chain that is required for the interpretation of the experimental result. Another big bottleneck is the simulation of the LHC detectors themselves—this is a more complicated problem and it relies, e.g., on empirical models. Nevertheless, many groups are working to address this problem mainly by leveraging generative machine learning techniques^5^.

A simple solution to speed up the computation of the functions is to build a regressor using a representative sample. However, to achieve the precision necessary for matching with experimental results, the regressors need to produce very high-accuracy predictions over the entire domain of the function. The requirements that we set for the regressors, and in particular what we mean by *high precision*, are:

- **High precision** =: = prediction error $< 1\%$ over more than $90\%$ of the domain of the function
- **Speed** $>:>$ prediction time per data point of $< 10^{-3}$ s
- **Lightweight** $>:>$ the disk size of the regressors should be a few megabytes at the most for portability

These requirements can be understood as follows. The prediction error, whose precise definition is given in Eq. (3), ensures that propagating the approximation error on $f_{02}$ to the full function *F* remains a sub-leading source of error, for example, compared with the Monte Carlo statistical error. Furthermore, the timing requirement ensures that evaluating $f_{02}$ is as fast as the next-to-slowest functions $f_{01}$ and $f_{11}$ thereby removing the bottleneck as we set out to do. Finally, the disk-size requirement is meant to ensure that even when multiple regressors are required, the total size remains small enough to allow any user regardless of their computing capability to perform a simulation using our regressors. Note also that this requirement favors neural networks over boosted decision trees (BDTs). In both cases, larger models are necessarily slower, and as such this requirement is not completely independent from the timing one.

The domain of the function of interest is called the phase space. Its coordinates comprise angular variables and energies. The former spans finite intervals while the latter does not, in principle. However, since no energy variable can take a value larger than the total energy of the collider by energy and momentum conservation, these variables effectively span a finite interval allowing us to set a precision requirement that spans a finite part of the domain. In this work, we explore the following concepts:

- With simulated data from real physics processes occurring in particle colliders, we study the error distributions over the entire input feature spaces for multi-dimensional distributions when using BDT, Deep Neural Networks (DNN), and Deep Neural Networks with skip connections (sk-DNN).
- We study these regressors for 2, 4, and 8 dimensional (D) data making comparisons between the performance of BDTs, DNN, and sk-DNNs with the aim of reaching errors smaller than 1–0.1% over at least 90% of the input feature space.
- We outline architectural decisions, training strategies, and data volume necessary for building these various kinds of high-precision regressors.

In what follows, we will show that we can reduce the compute time of the most compute-intensive part, $f_{11}( \user2{x} ) + f_{02}( \user2{x} )$ as defined in Eq. (1), by several orders of magnitude, down from several seconds to sub-milliseconds without compromising the accuracy of prediction. We show that physics-motivated normalization strategies, learning strategies, and invocation of physics symmetries will be necessary to achieve the goal of high precision. In our experiments, the BDTs outperform the DNNs for lower dimensions while the DNNs give comparable (for 4D) or significantly better (for 8D) accuracy at higher dimensions. DNNs with skip connections perform comparably with fully connected DNNs even with much fewer parameters and outperform DNNs of equivalent complexity. Moreover, DNNs and sk-DNNs meet and exceed the high-precision criteria with 8D data while BDTs fail. Our goal will be to make the most lightweight regressor for real-time prediction facilitating the speed-up of the Monte Carlo simulation.

## Related work

Building models for the regression of amplitudes has been a continued attempt in the particle physics literature in the recent past. In particular, BDTs have been the workhorse of particle physics for a long time but mostly for performing classification of tiny signals from dominating backgrounds^6^. However, the utility of BDTs as regressors for theoretical estimates of experimental signatures has only been advocated recently^7^ and has been shown to achieve impressive accuracy for 2D data.

Several other machine learning algorithms have been used for speeding up sample generation for Monte Carlo simulations. Ref.^8^ proposed the use of Normalizing Flows^9^ with Invertible Neural Networks to implement importance sampling^10,11^. Recently, neural network surrogates have been used to aid Monte Carlo Simulations of collider processes^12^. Reference^13^ used Bayesian Neural networks for regression of particle physics amplitudes with a focus on understanding error propagation and estimation. Reference^14^ attempted to reach the high-precision regime with neural networks and achieved 0.7% errors integrated over the entire input feature space while^15^ proposes to approximate multi-dimensional integrals via approximation with the addition of a proper correction term. And Ref.^16^ tackled parametric integrals, i.e., where only some of the variables are integrated over, that arise in precision particle physics calculations achieving 0.1–1% precision over a range of parameter values. Physics-aware neural networks were studied by Ref.^17^ in an attempt to handle singularities in the regressed functions. In the domain of generative models, GANs^18–20^, Normalizing Flows^21–23^ and VAEs^20^ have been used for sample generation^24,25^ in particular in connection with jet images and calorimeter simulations^26–33^.

Similar applications have surfaced in other domains of physics where Monte Carlo simulations are used. Self-learning Monte Carlo methods have been explored by Ref.^34^. Applications of Boltzmann machines^35^, deep neural networks^36^, and autoregressive neural networks^37^ have been seen recently. Reference^38^ use neural networks in Quantum Monte Carlo simulations to learn eigenvalues of Hamiltonians and the free energy of spin configurations, an application that lies outside the domain of particle physics. However, the primary goal of all these efforts has been to avoid first-principles computation and, hence, reduce compute time while staying below credible error budgets that are set in a problem-specific manner.

In contrast to prior works^7,8,24,25^, the novelty of our contribution is that we try to attain high precision in the entire domain of the function being regressed with fast and efficient regressors. For that, we compare BDTs and neural networks for functions with 2D, 4D, and 8D input feature spaces. We propose the use of a DNN with skip connections to avoid the problem of vanishing gradients for deeper neural networks and show that they perform better than fully connected DNNs. We also propose novel methods, derived from physics domain knowledge, for scaling the function being regressed with another function that is computationally inexpensive to calculate and is highly correlated with the function being regressed. We leverage the symmetry properties of the physical process under consideration for the reduction of the number of regressors required to be trained. The applicability of our work goes beyond the domain for which it has been developed and can be used for any application that requires high precision in speeding up simulations or sample generation.

## Physics context

The regressors we develop in this work will be used as surrogate models for exact functions that are numerically slow to evaluate. As a result of their (extreme) slowness, these exact functions, which are used in Monte Carlo simulations, are by far the biggest bottleneck in the simulation of the so-called hard process. As discussed in the introduction, this is only part of the full Monte Carlo simulation chain. However, unlike the detector simulation part which would only be performed in full by the experimental collaborations, hard-process generation is typically done by a wider community that includes theorists as well as experimentalists. Note that there are fast detector simulators such as Delphes^39^ which are used by theorists and experimentalists for development purposes.

The theoretical model that describes fundamental particles and their interactions is called the Standard Model of particle physics. The important feature of this theory is that computing observables—i.e., outcomes of experiments—cannot, in general, be done *exactly* because such calculations are not tractable for several reasons the explanation of which goes beyond the scope of this work. The usual way of obtaining predictions is by expanding the theory as a power series in a small expansion parameter and computing higher orders in this expansion to improve the accuracy of the prediction. Such perturbative expansions are ubiquitous in physics in general since only a few systems, most notably, e.g., the simple harmonic oscillator and the two-body inverse $r^2$ problem can be solved exactly. A very large fraction of physics problems spanning atomic physics, nuclear physics, condensed matter physics, astrophysics, cosmology, hydrodynamics, electrodynamics, quantum mechanics, complex systems, etc. requires the use of perturbative expansions where the higher order terms are very tedious and slow to compute. Hence, the methods we develop here are more broadly applicable in any problem where a perturbative expansion is used and/or a function that requires a very large number of evaluations is very slow to evaluate and a certain threshold of precision is required.

In this work, the functions $f_{ij}$ in Eq. (1) are the terms that arise in the production of four charged leptons in proton-proton collisions, $pp\rightarrow \ell _1^+\ell _1^-\,\ell _2^+\ell _2^-$, where each lepton pair is mediated by an electrically neutral electroweak gauge boson, i.e., a *Z* boson or a photon. For this process, the leading-order function, $f_{00}$, corresponds to the square of the tree-level scattering amplitude. A tree-level amplitude is associated with graphs that do not contain internal legs that form a closed loop while higher terms have graphs that do. For example, the time penalty for improving the accuracy of the prediction of the rate of production of four electrons by including the second-order term is a factor of 1500. For details, please see Table 11 in the journal version of Ref.^40^.

Note that while we focus on the second-order term in this work, the methods we develop here apply equally to higher-order terms. In particular, our focus here is on functions of the form $f_{ij}$ where $i+j\ge 2$ and $\max \{i,j\} >1$ (functions with $i,j\le 1$ are typically fast enough, though there are exceptions). These functions involve scattering amplitudes the largest number of loops multiplying the tree-level ones, hence the zero subscript ‘0’. For this reason, these functions have graphs with the lowest number of external legs and, correspondingly, have the lowest possible domain dimensionality for a given process. Here we focus on $pp\rightarrow \ell _1^+\ell _1^-\ell _2^+\ell _2^-$ which has six external particles, i.e., six external legs. Such a process has an eight-dimensional (8D) domain. Furthermore, because the leptons, $\ell _1^\pm$ and $\ell _2^\pm$, are mediated by electroweak gauge bosons, the full process can be decomposed into one subprocess with four legs and two with three legs; this leads to functions with 4D domains. Finally, to obtain ones with 2D domains, we take both electroweak gauge bosons to be *Z* bosons and fix their masses. This is just a special case used here for comparison purposes only. A realistic simulation would rely on the 4D or 8D functions.

### Physics simulations

The functions in question are maps, $f^{(n)}_{ij}\equiv f_{ij}(x_1,\ldots ,x_n):\mathbb {R}^n\rightarrow \mathbb {R}$, where $n\in \{2, 4, 8\}$ and $i,j\in \{0,1,2\}$, cf. (1). The domain of the functions, i.e. the feature space, is linearly mapped to the unit hypercube and populated from a uniform distribution. For example, the physical phase-space coordinates in the 4D case are: the total energy of the process, $\sqrt{s_{12}}$, the scattering angle of the di-boson system, $\theta ^*$, and the masses of the two bosons, $m_{34}$ and $m_{56}$. These masses are not in general fixed because the bosons are said to be off their mass shell. In the 8D case, there are additional angles associated with the leptons in the final state, and in the 2D case, the masses are fixed. The physical phase-space variables are linearly mapped to the unit hypercube—their physical ranges are:2

The corresponding datasets are generated using the particle physics simulation code VVAMP^41^ from first principles using building-block functions that we will refer to as form factors. Apart from the 2D dataset, which is a special case of the 4D one, the same form factors were used to generate the 4D and 8D datasets. The difference between the 4D and 8D feature spaces lies in the physics of the process in question, namely the number of external particles the functions describe. The regressor of the 4D functions, $g^{(4)}_{ij}\approx f^{(4)}_{ij}$, can be used to generate the 8D functions, $f^{(8)}_{ij}$, after multiplying by two other (exact) functions that are computationally inexpensive to calculate and summing them.

The number of resulting functions, technically called helicity amplitudes, depends on the dimension as shown in Table 1. While the number of required regressors for the 4D feature space is the largest, it also offers the most flexibility for downstream physics analyses. To generate the 8D functions, more details of the process have to be specified during data generation which is then frozen into the regressor. Consequently, different physics analyses will require different regressors. By contrast, the 4D regressors are more general-purpose and do not contain *any* frozen physics parameters.

**Table 1:** The number of total and independent functions that arise at 2, 4, and 8D and the number of independent functions, a minimal subset from which the other functions can be generated by re-mapping the feature space.

Symmetry properties reduce the number of required functions			
Dimensionality	Total functions	Independent functions	Sum is physical?
2D	18	5	Yes
4D	162	25	No
8D	8	4	Yes

The smaller number of necessary functions in the third column of Table 1 is obtained by leveraging the symmetry properties discussed below derived from physics domain knowledge. For the data used in this analysis, it stems from the symmetries manifest in the scattering process that was simulated. The last column indicates whether summing the functions has a physics meaning; in the cases where it does, i.e. 2D and 8D, only the single regressor of the sum of the functions is required.

**Symmetry properties** The full set of functions, $f^{(n)}_{ij}$, for any dimension, *n*, is over complete. Pairs of functions can be mapped into one another via particular permutations of the external particles the process describes. This translates into a linear transformation on the second coordinate, $x_2$, independently and in combination with the permutation of the third and fourth coordinates, $x_3$ and $x_4$, in feature space. For example, in 4D, two permutations $\pi _{12}:p_1\leftrightarrow p_2$ and $\pi _{34}:=p_3\leftrightarrow p_4$, where $p_i$ is a particle with label *i* reduces the number of independent functions from 162 to 25.

**Table:** 

Permutation	Particle symmetry	Coordinate symmetry
$\pi _{12}$	$p_1\leftrightarrow p_2$	$x_2 \rightarrow 1-x_2$
$\pi _{34}$	$p_3\leftrightarrow p_4$	$x_2 \rightarrow 1-x_2$ and $x_3\leftrightarrow x_4$

**Computational burden of Monte Carlo simulations** Generating the 2D, 4D and 8D datasets required 144 hours on 96 AMD EPYC 7402 cores for 13 million data points per set. This had to be done twice, once for the 2D dataset and once for the 4D and 8D datasets which were generated from the same computationally intensive form factors that have to be calculated from first principles. In contrast, the regressors that we build generate a million samples in a few seconds to a few minutes on any desktop computer.

## Models: decisions trees and neural networks

In this section, we will develop several methods that will enable us to achieve the high-precision requirements that we set. As a benchmark, we will use the condition: $|\delta |<$ 1% in over 90% of the domain of the function being regressed (for a more detailed explanation of the precision requirements please read “Physics context”) Here, $\delta$ is defined as the difference between the predicted value of the function, $f(\user2{x})_{{{\text{predicted}}}}$, and its true value, $f( \user2{x} )_{true}$, normalized by $f( \user2{x} )_{true}$, or,

3 $$\delta = \frac{{f(\user2{x})_{{{\text{predicted}}}} - f(\user2{x})_{{{\text{true}}}} }}{{f(\user2{x})_{{{\text{true}}}} }}$$

knowing a priori that $f(\user2{x})_{{{\text{true}}}}$ is positive definite. Usually, the performance of a regressor and model comparison in machine learning is done using a single accuracy measure which is a statistical average of the distribution for that accuracy measure over the entire test sample. This, however, does not provide a complete picture of the accuracy of the regressor in high-precision applications. Using error distributions instead of a single number leads to a better criterion for model selection as the tails of the error distribution can then be examined for abnormally large errors in a small part of the parameter space. Hence, in addition to quoting statistics of the error distributions we also show the error distributions in the “Results” section.

**Physics informed normalization** An attempt to build regressors with the raw data from the Monte Carlo simulations results in a failure to meet the high-precision requirements that we have set. Hence, we have to appeal to a novel normalization method derived from the physics that governs the physical processes. The functions of interest in particle physics processes at colliders are often very highly peaked in one or more dimensions. This makes it quite difficult to build a regressor that will retain the desired high precision over the entire domain of the function. This problem cannot be addressed by log scaling or standardizing to zero mean and unit variance since the peaks can be quite narrow and several orders of magnitude greater than the mean value of the function. A regressor trained on the log-scaled function provides an error distribution over the entire domain which, when exponentiated, transforms to large errors around the peak. This behavior is not desirable. Normal scaling does not help since the standard deviation of the distribution is much smaller than the peak value, often being several orders of magnitude smaller, making the peak values outliers.

**Figure 1:**
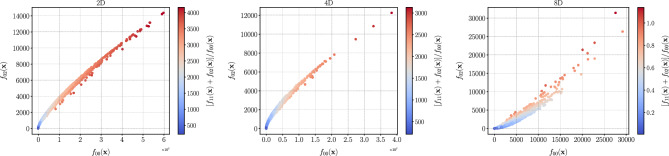
The effects of normalizing second-order term $f_{02}( \user2{x} )$, with the zeroth-order term, $f_{00}( \user2{x} )$. The two functions are highly correlated ($\rho = 0.87$ for 8D, $\rho = 0.91$ for 4D and $\rho = 0.96$ for 2D) and the resulting normalized functions, $f( \user2{x} ) = [f_{11}( \user2{x} ) + f_{02}( \user2{x} )]/f_{00}( \user2{x} )$ are much less peaked.

As a solution, we normalized the second-order contribution with the zeroth-order contribution as defined in Eq. (1), i.e., we transform to a distribution:

4 $$\begin{aligned} f( \user2{x} ) = \frac{f_{11}( \user2{x} ) + f_{02}( \user2{x} )}{f_{00}( \user2{x} )}\,, \end{aligned}$$

where $f( \user2{x} )$ is the function that will be regressed. This first-order term, $f_{00}( \user2{x} )$, also has a peak similar to and is highly correlated with the second-order term, $f_{11}( \user2{x} ) + f_{02}( \user2{x} )$, with $\rho \sim 0.9$. Hence, this normalization yields a distribution, $f( \user2{x} )$, that is more tractable to regress. We show examples in Fig. 1 where one can see that both $f_{00}( \user2{x} )$ and $[f_{11}( \user2{x} ) + f_{02}( \user2{x} )]$ are both very peaked and span several orders of magnitude but their ration spans only one order of magnitude as the two terms are highly correlated. Computation of the first-order term from first principles is numerically inexpensive and does not require regression. Furthermore, we standardize the distribution by removing the mean and scaling to unit variance.

### DNN design decisions

The DNN architectures that we used are fully connected layers with *Leaky ReLU*^42^ activation for the hidden layers and linear activations for the output layer. We show a comparative study of activation functions in “Deep neural networks and skip connections” where we find that the *Leaky ReLU* outperforms other activation functions like *ReLU*^43,44^, *softplus* and *ELU*^45^. We do not consider any learnable activation functions in this work and leave it for a future work. To assess progress during the training stage, we compute the coefficient of determination, $R^2$, which is defined via,

5 $$1 - R^{2} = \frac{{\sum\nolimits_{i} {(f(\user2{x}_{i} )_{{{\text{predicted}}}} - f(\user2{x}_{i} )_{{{\text{true}}}} )^{2} } }}{{N{\mkern 1mu} \sigma ^{2} }}{\mkern 1mu} ,$$

where $\sigma ^2$ is the variance of the true values over the validation set of *N* points labeled by the index *i*. Note that $1-R^2$ is proportional to our chosen objective function (the mean-squared error). It is a useful figure of merit during the training phase because the constant of proportionality, $\sigma ^2$, standardizes it in a way that allows us to self-consistently compare different models, while the mean-squared error itself does not. To fully characterize the performance of the models, however, we will define later, in “Model comparison”, a figure of merit, $R_\tau$, which specifies the percentiles of relative errors greater than 1% and 0.1%. This allows us to more fully characterize the *distribution* of relative approximation errors and has a precise meaning given a particular error probability density function, see Fig. 6. We use the following design decisions:

**Objective function** We use the mean-squared-error loss function without any explicit regularization in the objective function. While we use linear relative error, $\delta$, to estimate the performance of the model over the entire feature space, our decision to use the mean-squared-error loss function is made so as to preferentially penalize outliers and reduce their occurrence. The data we use is noise-free as they are generated by ab initio computation of the functions. Secondly, we use very large datasets to avoid data sparsity in any part of the domain. Lastly, we use an early stopping condition to avoid the low bias/high variance regime which acts as an implicit regularizer. These together imply that explicit regularization in the objective function is not necessary as overfitting is sufficiently mitigated and the required generalization is achieved. Increasing the capacity of the models beyond what we use ultimately leads to overfitting, especially for 8D data and we observe that. Hence, we do not allow the models to have a higher capacity than necessary. Furthermore, we avoid underfitting by increasing the capacity of the models to move out of the low variance/high bias regime.

**Learning rate** It is necessary to cool down the learning rate as a minimum of the objective function is approached. This is absolutely necessary to search out an optimum that allows for uniformly low error over the entire feature space. For both the DNN and the sk-DNN, we use the Adam optimizing algorithm. Ref.^46^ discuss an inverse square-root decay rate scaling for the Adam optimizer. We do not find this optimal for this high-precision application. The square-root cooling is quite rigid in its shape as it is invariant to scaling up to a multiplicative constant. Hence, we use an exponential cooling of the learning rate which has an asymptotic behavior similar to the inverse-square-root scaling but its shape is far more tunable. The learning rate is cooled down starting from $10^{-3}$ at the beginning of the training to $10^{-6}$ at 2500 epochs. Much of the learning is completed during the early stages of the training, i.e. within 200 epochs.

The $R^2$ score at this point is about 0.5% from the final $R^2$ score (> 99.9%). However, to attain the high-precision requirements, the final stages of the training are necessary and take about 2500–3000 more epochs.

**Training epochs and validation** An early stopping criterion based on RMSE is used to determine the number of epochs the regressors is trained for with *patience* set to an unusually large number, 200 epochs. We define patience as the number of epochs after which the training is stopped as no reduction is seen in the RMSE computed from the validation set and the weights and biases are reset to those corresponding to the lowest RMSE. We use this large patience to allow the optimizer to possibly move to a better optimum while having a very small learning rate if a better one exists. We first split the data into 20% test set and 80% training and validation set. The latter set is further split into 60% training set and 40% validation set. This results in a 20%–48%–32% split for test, train, and validation respectively. The large validation set is necessary to make sure that errors are uniformly low over the entire domain of the function being regressed. For all cases, we use a dataset with 10 million samples.

**Weight initialization** For both the DNN and the sk-DNN, the weight matrices are initialized using Glorot initialization^47^. We also checked uniform and Gaussian initializations and concluded that Glorot initialization works best for our models.

**Other hyperparameter tunings** We explore network architectures of various depths and widths for both the DNN and sk-DNN. We show results for three representative architectures only, including the best-performing one for each neural network type. Increasing the depth and/or width beyond the best architectures shown in the “Results” section did not provide any gain in accuracy and, after a point, showed slightly lower performance. Moreover, wider and deeper architectures take longer to train without any performance gain. We also scan batch size and steps per epoch and set them to 512 and 4000 respectively.

### DNN with skip connections

**Figure 2:**
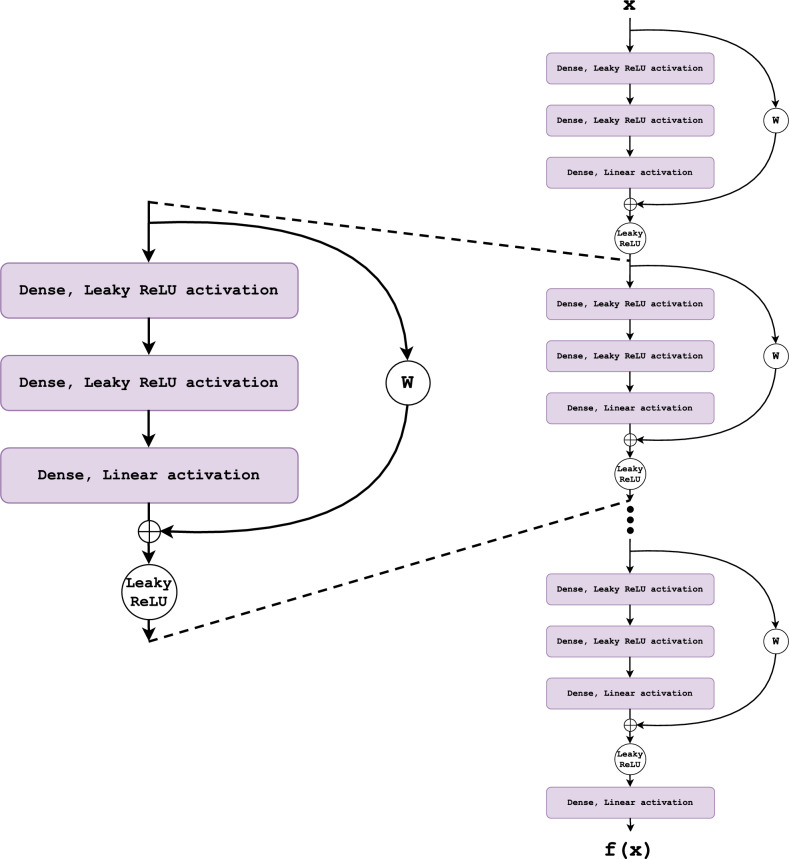
The building block for a DNN with skip connections. The first two layers are fully connected with *Leaky ReLU* activation. The last layer has linear activation and is added to the input through the skip connection before being transformed with a non-linear *Leaky ReLU* function. The matrix $\user2{W}$ is a weight matrix that is trainable if the input and output dimensions are different for the block and $\user2{I}$ otherwise. The blocks are stacked sequentially to form the neural network.

In addition to a fully connected DNN, we also experiment with a DNN with skipped connections (sk-DNN) to address the problem of vanishing gradients for deeper neural networks^48–52^. The building block of the sk-DNN is illustrated in Fig. 2. Given an input $\user2{x}$ the output of the block is

6 $$\begin{aligned} \user2{y}= g\left( h( \user2{x} ) + \user2{W} \user2{x} \right) \end{aligned}$$

where $h( \user2{x} )$ is the output of the third layer with linear activation, *g* is a non-linear function and $\user2{W}$ is a trainable weight matrix of dimension $i\times j$ when the input dimension, *i*, is different from the output dimension, *j*, and $\user2{I}$ otherwise. The structure of this block can be derived from the Highway Network^48^ architecture with the *transform* gate set to $\user2{I}$ and the *carry* gate set to $\user2{W}$ for $\dim ( \user2{x} )\ne \dim (\user2{y})$ and $\user2{I}$ otherwise. Structurally, the sk-DNN block is similar to a ResNet block^49^ with a different set of hidden layers.

We keep the normalization of the target variable and the learning rate decay schedule the same as for the DNN. We also test the sk-DNN with the weight matrix, $\user2{W}$ fixed with a random initialization of the elements and see no difference in the accuracy of the model and hence keep $\user2{W}$ trainable. The lack of any difference is because the number of trainable weights in the first block amount to the dimension of the input vector and hence are negligible compared to the number of total parameters of the models.

## Results

We proceed to derive the optimal hyperparameters for the models that will facilitate the high-precision requirements. We also study a set of activation functions to best design the neural network architectures. With these, we provide a comparison of the accuracy of all the models for all the datasets.

### Boosted decision trees

**Figure 3:**
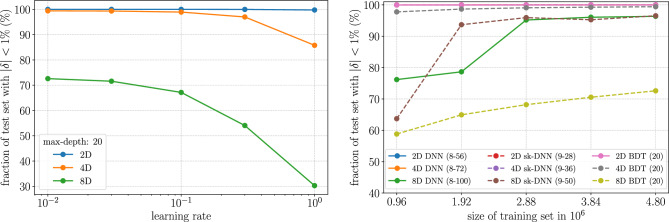
Left panel: the effect of learning rate on achieving high precision with BDTs. The learning rate is not an important parameter for low dimensions but is significant for higher dimensions. Right panel: the data volume required to train the different regressors. For lower dimensions small volumes of data ($< 1$M) are sufficient. However, for higher dimensions, a lot more data is necessary.

We use XGBoost^53^ to implement the BDTs. In varying the architecture of the regressors, we focus on the *max-depth* of the BDT which is a hyperparameter that controls the maximum depth to which a tree grows in a boosted ensemble. If Fig. 3 we show how changing the learning rate and the training data volume changes the accuracy of the trained BDT models. In the final version of our experiments, we use a learning rate of 0.01 and 10 million data points of which 48% is used for training, 32% is used for validation and early stopping and 20% is used for testing. More details on hyperparameter correlation and selection can be found in “Hyperparameter surveys for boosted decision trees”.

### Hyperparameter surveys for boosted decision trees

**Figure 4:**
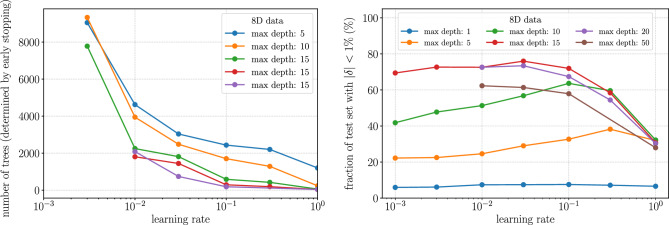
Left panel: The variation of the number of trees grown in a BDT ensemble increases rapidly with decreasing learning rate when using an early-stopping criterion. Note: The learning rate for BDTs with maximum depths 20 and 50 could not be reduced below 0.1 as the disk size of the memory consumption while training the models with 8D data gets too large for a single node in a high-performance computing cluster. Right panel: Larger maximum depth for BDT ensembles gives better accuracy up until a certain value and then the accuracy falls. The optimal value for maximum depth seems to be 15 or 20. Also, the learning rate has an optimal after which it decreases or plateaus.

**Maximum depth of trees in the ensemble** The BDT models are trained with an early stopping condition which stops the growth of the trees once the RMSE stops decreasing after checking for its decrease for 25 rounds. This makes the hyperparameters used to train a BDT correlated to a certain extent. For example, a decrease in the learning rate increases the number of trees grown till the optimum is reached. This can be seen from Fig. 4. However, as one increases the maximum depth to which each tree can grow the number of total trees grown decreases. The number of nodes of a tree grows exponentially with the depth of the trees and, hence, allowing for a larger maximum depth of the trees results in a much larger disk size for the trained models. This is aggravated further with higher dimensional data. Therefore, when portability is a concern, BDTs cannot be used for high-precision applications for higher dimensional data.

**Learning rate and maximum depth of trees** When exploring the learning rate for the BDT models in Fig. 4, we find that, initially, with decreasing learning rate, starting at 1, the accuracies of the trained models increase but after a point, the accuracy decreases. This is evident for shallower trees. We also note that the accuracies of the models increase with the maximum depth of the trees but only up to a certain depth. In the example in the right panel of Fig. 4 we use the 8D data and we see that the accuracy of the model increases till a maximum depth of 15 and then decreases. In addition, we had to take into consideration the increasing disk size of the models for deeper trees. Hence we did not build BDTs of depth greater than 50 as their disk size would already be greater than a few hundred megabytes even for the 2D and 4D datasets (and a few gigabytes for the 8D dataset) for very marginal gains in accuracy.

We performed a grid search for a few other parameters like “column sample by tree” (the subsample ratio of columns when constructing each tree) and “subsample (the fraction of the training sample used for growing a tree)”. These two proved to be optimal at their default value of 1.

### Deep neural networks and skip connections

We implemented all neural network architectures with TensorFlow^54^. For the neural networks, we focus on the depth, width and number of trainable parameters in the regressor (denoted as width-depth, with trainable parameters in parentheses, in the tables and figures). The depth of the sk-DNN denotes the number of sequential sk-DNN blocks in the regressor and not the total number of layers. The width of the sk-DNNs is chosen to be half the width of the DNNs and the depth of the sk-DNN is adjusted so that they have approximately the same number of parameters as the DNNs with similar depth. An sk-DNN with 2 blocks is an exception and has more parameters than the corresponding DNN with 2 layers. The data strategy remains the same as for the BDTs.

**Figure 5:**
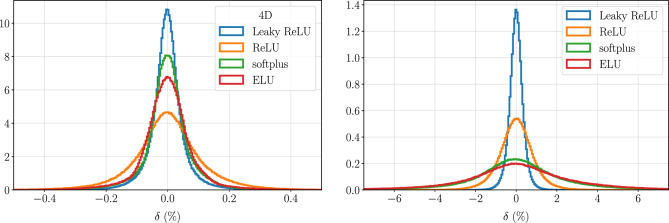
A comparison between *Leaky ReLU*, *ReLU*, *softplus* and *ELU* using an sk-DNN (left panel:) for 4D with 9 blocks of width 36 and (right panel:) for 8D with 9 blocks of width 50. The *Leaky ReLU* activation function outperforms any other activation function and this is more prominent at 8D than at 4D. We use it for all experiments with DNNs and sk-DNNs in our work.

We performed tests for various activation functions keeping all other hyperparameters and data strategies the same. We use the 4D and 8D datasets with a 9-deep and 36-wide sk-DNN for 4D and 9-deep and 50-wide sk-DNN for 8D on an exponential learning rate schedule and data normalized using Eq. (4). We explore only non-trainable activation functions like the *ReLU*, *Leaky ReLU*, *ELU* and softmax activations functions. The last three were chosen as they are similar to *ReLU* and have shown improved learning abilities in several domains^42,44,45^. As in the other experiments, the models were trained with an early-stopping criterion. From  Fig. 5 we see that the *Leaky ReLU* activation function far outperforms all other activation functions with a narrower error distribution. This is more prominent for 4D data than for 8D data. Hence, for all experiments in this work, we use the *Leaky ReLU* activation function.

### Model comparison

To compare the performance of the regressor we use the distribution of $\delta$ (defined in Eq. (3)). We focus on this distribution as it is important for the high-precision requirement to identify the fraction of test data that has large errors. We will identify the following statistics:

- $R_{\tau }$: the fraction of the test set that has $|\delta |$ less than $\tau$%
- $\mu _\delta$: the mean of the $\delta$ distribution
- $\sigma _\delta$: the standard deviation of the $\delta$ distribution

**Figure 6:**
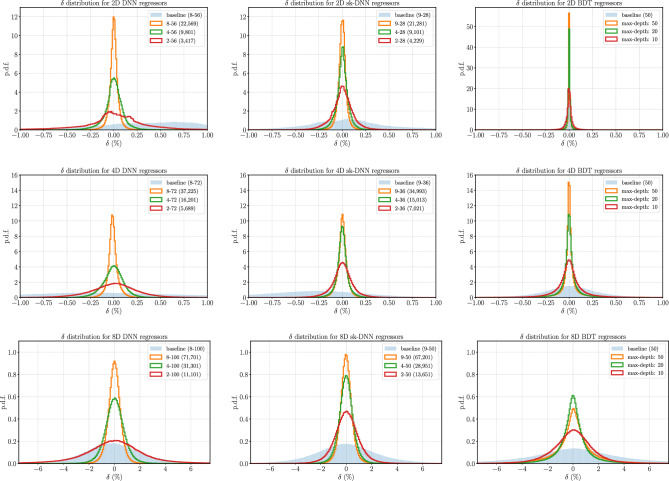
The $\delta = (f(\user2{x})_{{{\text{predicted}}}} - f(\user2{x})_{{{\text{true}}}} )/f(\user2{x})_{{{\text{true}}}}$ distribution for the various 2D (upper panels), 4D (middle panels), and 8D (lower panels) regressors. The labels for the DNN and sk-DNN designate *depth-width (number of parameters)* where depth is the number of layers for the DNN and the number of blocks for the sk-DNN. For the BDTs, the labels denote *max_depth:* *N* with max_depth being the maximum depth of the trees. In each panel, we compare three models with increasing complexity—i.e., with a larger number of parameters for the neural networks and max_depth for the BDTs—to a baseline model whose definition is given in “Model comparison”. The area under each error distribution histogram is normalized to unity.

**Baselines** To understand the efficacy of the optimization strategies that we developed, we build a baseline without any optimization for BDTs, DNNs and sk-DNNs. We do not normalize the data as described in “Models: decisions trees and neural networks”, rather, we only log scale the data. We set the train-validation split to 80–20%. For the BDTs, we use an ensemble with max-depth = 50 and set the learning rate to 0.1. For the DNNs and sk-DNNs, we fix the learning rate of the Adam optimizer at $10^{-3}$, lower the patience to 10 rounds, and use the most effective architecture chosen from amongst the high-precision regressors. The results are presented in Table 2 and Fig. 6 and clearly display the effects of the optimizations used.

**Table 2:** Parameters extracted from the error distributions shown in Fig. 6. Predictions from 1 million test samples were used to generate these statistics. The best-performing models for each of 2D, 4D, and 8D are marked in bold. The notation for the DNN and sk-DNN regressors is *depth-width (number of parameters)* where *depth* is the number of layers for the DNN and the number of blocks for the sk-DNN. For the BDTs, the labels denote *max_depth: N* which is the maximum depth of the trees. The third and fourth columns show the fraction of the test dataset with $|\delta |<1\%$ and $|\delta |<0.1\%$, respectively. The second-to-last and last columns give the mean and the standard deviation of the distribution of errors, $\delta$, for the test set.

	2D	$R_{1} (\%)$	$R_{0.1} (\%)$	$\mu _{\delta } (\%)$	$\sigma _{\delta }(\%)$
DNN	2–56 (3417)	95.54	32.43	0.0114	0.52
	4–56 (9801)	99.95	75.58	$-0.0005$	0.13
	8–56 (22,569)	99.99	94.43	$-0.0$	0.06
	Baseline (8–56)	77.16	9.01	0.1361	1.83
sk-DNN	2–28 (4229)	99.92	67.71	0.0001	0.14
	4–28 (9101)	99.99	87.95	0.0012	0.07
	9–28 (21,281)	100.0	95.72	-0.0005	0.05
	Baseline (9–28)	90.79	15.31	0.0173	1.39
BDT	Max-depth: 10	100.0	95.01	-0.0001	0.05
	Max-depth: 20	100.0	99.1	0.0	0.03
	**Max-depth: 50**	**100.0**	**99.16**	**0.0**	**0.02**
	Baseline (50)	99.91	94.04	$-0.0045$	0.1

	4D				
DNN	2–72 (5689)	99.18	34.34	0.002	0.32
	4–72 (16,201)	99.97	66.42	$-0.0068$	0.13
	8–72 (37,225)	100.0	91.58	$-0.0096$	0.07
	Baseline (8–72)	88.67	13.63	0.0449	1.1
sk-DNN	2–36 (7021)	99.96	69.23	0.0004	0.13
	4-36 (15,013)	100.0	89.24	$-0.0009$	0.07
	**9–36 (34,993)**	**100.0**	**92.85**	**0.0007**	**0.06**
	Baseline (9–36)	84.99	10.81	0.2701	1.11
BDT	Max-depth: 10	99.26	66.16	0.0014	0.22
	Max-depth: 20	99.41	81.34	0.0016	0.18
	Max-depth: 50	99.4	83.19	0.0017	0.18
	Baseline (50)	95.85	27.8	0.0023	0.55

	8D				
DNN	2–100 (11,101)	37.2	3.95	0.1322	4.13
	4–100 (31,301)	82.37	11.64	0.029	1.05
	8–100 (71,701)	94.22	18.12	0.0016	0.6
	Baseline (8–100)	31.97	3.3	0.799	4.38
sk-DNN	2–50 (13,651)	72.76	9.31	0.049	1.5
	4–50 (28,951)	90.98	15.69	0.0035	0.7
	**9–50 (67,201)**	**94.94**	**19.36**	**− 0.0063**	**0.56**
	baseline (9–50)	30.91	3.23	$-0.547$	4.85
BDT	Max-depth: 10	51.68	5.95	0.0921	2.91
	Max-depth: 20	72.6	12.06	0.0577	1.85
	Max-depth: 50	62.15	9.61	0.1505	2.36
	baseline (50)	22.33	2.3	1.3953	13.35

**Figure 7:**
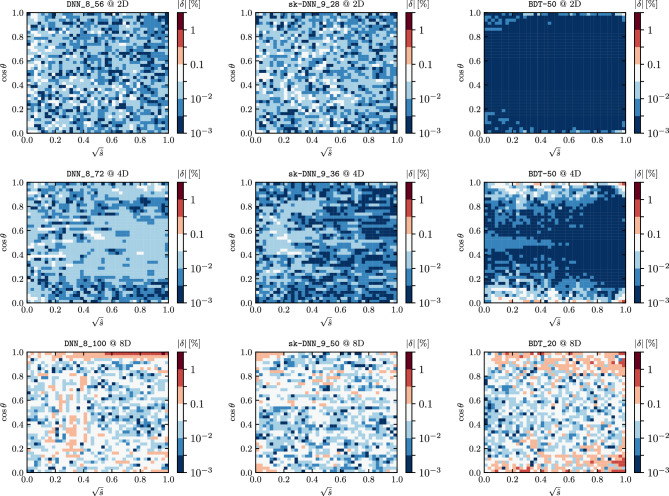
The $\delta = (f(\user2{x})_{{{\text{predicted}}}} - f(\user2{x})_{{{\text{true}}}} )/f(\user2{x})_{{{\text{true}}}}$ distribution for the various 2D (upper panels), 4D (middle panels) and 8D (lower panels) regressors. The errors are averaged over each bin.

**Key results** we present the results of the experiments in Table 2 and Fig. 6. We show the distribution of errors over two variables, square root of the center-of-mass energy, $\sqrt{s}$ $\mapsto x_1$, and $\cos \theta$ $\mapsto x_2$ in Fig. 7. It is clear that the BDTs far outperform the DNNs in 2D. However, at 4D and 8D the sk-DNN not only outperforms the fully connected DNNs, but also outperforms the BDTs as can be seen from the distributions in Fig. 6 and the numbers in Table 2. While at 4D the improvement of accuracy from the DNN and sk-DNN is marginal over the BDTs, at 8D the improvement of accuracy is quite significant. One major disadvantage of the BDTs is that they take up significant disk space as the ensemble grows large, especially at higher dimensions, which is necessary for high-precision applications but affects their portability. Hence the sk-DNNs are a good solution for having a portable, yet accurate regressor that meets the thresholds we set at the beginning of the work.

Besides the optimized hyperparameters for the BDTs, the normalization of the data that we perform is the primary source of improved performance of the models. The reason behind this is that the transformation done to normalize the data is non-monotonic and, hence, affects the performance of the BDTs. For the neural networks, in addition to the improvement in performance because of the data normalization, defining a proper learning schedule and enforcing a very strict early stopping criterion are also equally important for achieving the requisite performance.

## Conclusion

With Monte Carlo simulation in Physics being time and resource-intensive, a distinct necessity of building regressors for speeding up the simulations exists. We carefully examine the requirements of high precision for these regressors and lay down design strategies to achieve the necessary benchmarks. We use domain knowledge from particle physics to determine normalization strategies, apply symmetry arguments to reduce the number of necessary regressors, and set benchmarks for high-precision regression.

We show that boosted decision trees are reliable workhorses that can easily outperform DNNs at lower dimensions even when very large and complex neural networks are used. However, this edge that BDTs have over neural networks tends to fade at higher dimensions especially when DNNs with skip connections are used. In fact, for 4D and 8D data, sk-DNNs outperform both DNNs and BDTs and exceed the benchmark of $|\delta | < 1\%$ over $90\%$ of the domain of the function. Moreover, sk-DNNs are capable of outperforming DNNs of higher complexity as can be seen from  Table 2.

The primary disadvantage of BDTs is that for higher dimensions the ensemble of trees grows large enough to take a significant amount of disk space, often $>1$ GB, affecting the portability of the regressor if it is intended to be used as part of a Monte Carlo simulation package. On the other hand, the disk space occupied by a neural network stays below a few megabytes, making them a lot more portable. In summary, the important conclusions of our work are:

- High precision regressors required to speed up Monte Carlo simulations by factors of $10^3$–$10^6$ are better optimized by leveraging physics domain knowledge and symmetry arguments.
- BDTs outperform DNNs at lower dimensions but start to make large errors in predictions in parts of the function domain at higher dimensions. While fully connected DNNs perform relatively well at higher dimensions, a DNN with skip connections outperforms both BDTs and fully connected DNNs at 4D and 8D.
- sk-DNNs can outperform DNNs with a larger number of parameters.
- Compared to the few seconds that it takes for a single sample generation during a Monte Carlo simulation, the regressors we design can provide precise predictions in milliseconds to microseconds.

We have not explored pruning and knowledge distillation of networks or pruning of BDTs as methods for reducing the number of model parameters and the model size^55,56^. For neural networks, we achieve the desired accuracy and model size requirements and hence making them smaller is not necessary. For boosted decision trees, pruning cannot reduce model size to a few megabytes while retaining the desired accuracy for our use case.

In this work, we aimed at reaching the desired precision but by no means have we exhausted the possibilities of achieving even higher precision. As future directions, surveying a wider gamut of activation functions, the modifications of the likelihood with possible physics constraints or symmetry arguments or further reducing the number of models by simultaneously predicting a set of functions from a single neural network might be directions that can be explored in detail.

### Contribution to sustainability

Monte Carlo simulations of physics processes leave a very large carbon footprint. Our work directly addresses one of the contributors to the total simulation CPU time, namely, event generation. For the ATLAS experiment, for example, event generation consumes 11%^57^ of the total simulation CPU time and the projection for 2031 with aggressive R &D is 20%^58^. In addition, Monte Carlo simulators are ubiquitous in phenomenological analyses. Hence, our work directly contributes to reducing the carbon footprint significantly through a much more efficient way of generating these events.

Generating the 2D, 4D and 8D datasets required 144 hours on 96 AMD EPYC 7402 cores for 13 million data points per set. This had to be done twice, once for the 2D dataset and once for the 4D and 8D datasets which were generated from the same first principles computation. In contrast, the regressors that we build generate a million samples in a few seconds to tens of seconds on any desktop computer. The regressors we build can be trained on personal computers with a few CPU threads and a single GPU in about a day as our focus has been to build lightweight models. No special hardware is required to train or test these regressors. Given that these Monte Carlo simulations have to be done thousands of times during the life cycle of a single analysis, including distributed efforts for phenomenological analyses and any other exploratory analyses, the regressors can significantly reduce the carbon footprint from energy consumption without any significant compromise to the precision necessary for quantitative scientific research.

## Data Availability

Code and data necessary to reproduce this work are available at https://github.com/talismanbrandi/high-precision-ml.

## References

[1] LHC Design Report Vol. 1: The LHC main ring. 10.5170/CERN-2004-003-V-1 (2004).

[2] Aad G (2008). The ATLAS experiment at the CERN large Hadron Collider. JINST.

[3] Chatrchyan S (2008). The CMS experiment at the CERN LHC. JINST.

[4] Gianotti, F. *et al*. Physics potential and experimental challenges of the LHC luminosity upgrade. *Eur. Phys. J. C*. **39**, 293–333. 10.1140/epjc/s2004-02061-6 (2005)

[5] Adelmann, A. *et al*. New directions for surrogate models and differentiable programming for High Energy Physics detector simulation. in *Snowmass 2021* (2022). eprint2203.08806.

[6] Radovic A (2018). Machine learning at the energy and intensity frontiers of particle physics. Nature.

[7] Bishara F, Montull M (2023). Machine learning amplitudes for faster event generation. Phys. Rev. D.

[8] Winterhalder, R. et al. Targeting multi-loop integrals with neural networks. SciPost Phys. 12, 129. 10.21468/SciPostPhys.12.4.129 (2022).

[9] Jimenez Rezende, D. & Mohamed, S. Variational inference with normalizing flows. arXiv e-prints (2015). eprint1505.05770.

[10] Müller, T., McWilliams, B., Rousselle, F., Gross, M. & Novák, J. Neural importance sampling. CoRR e-prints (2018). eprint1808.03856.

[11] Ardizzone, L. *et al*. Analyzing inverse problems with invertible neural networks. CoRR e-prints (2018). eprint1808.04730.

[12] Danziger, K., Janßen, T., Schumann, S. & Siegert, F. Accelerating Monte Carlo event generation – rejection sampling using neural network event-weight estimates. *SciPost Phys*. **12**, 164. 10.21468/SciPostPhys.12.5.164 (2022).

[13] Badger, S., Butter, A., Luchmann, M., Pitz, S. & Plehn, T. Loop Amplitudes from Precision Networks. arXiv (2022). eprint2206.14831.

[14] Chen, I.-K., Klimek, M. D. & Perelstein, M. Improved neural network Monte Carlo simulation. *SciPost Phys*. **10**, 023. 10.21468/SciPostPhys.10.1.023 (2021).

[15] Yoon, B. A machine learning approach for efficient multi-dimensional integration. *Sci. Rep*. **11**, 18965. 10.1038/s41598-021-98392-z (2021). 10.1038/s41598-021-98392-z PMC8460840 34556754

[16] Maître, D. & Santos-Mateos, R. Multi-variable Integration with a Neural Network. arXiv e-prints (2022). eprint2211.02834.

[17] Maître D, Truong H (2021). A factorisation-aware Matrix element emulator. JHEP.

[18] Goodfellow, I. J. *et al*. Generative adversarial networks. arXiv e-prints (2014). eprint1406.2661.

[19] Springenberg, J. T. Unsupervised and semi-supervised learning with categorical generative adversarial networks. 4th International Conference on Learning Representations, ICLR 2016, San Juan, Puerto Rico, May 2-4, 2016, Conference Track Proceedings (2016). arXiv:1511.06390.

[20] Brock, A., Donahue, J. & Simonyan, K. Large scale GAN training for high fidelity natural image synthesis. CoRR e-prints (2018). eprint1809.11096.

[21] Tabak EG, Vanden-Eijnden E (2010). Density estimation by dual ascent of the log-likelihood. Commun. Math. Sci..

[22] Tabak, E. G. & Turner, C. V. A family of nonparametric density estimation algorithms. *Commun. Pure Appl. Math*.**66**, 145–164. 10.1002/cpa.21423 (2013)

[23] Rezende, D. J. & Mohamed, S. Variational inference with normalizing flows. in *Proceedings of the 32nd International Conference on International Conference on Machine Learning*-Volume 37, ICML’15, 1530-1538 (JMLR.org, 2015).

[24] Butter, A., Diefenbacher, S., Kasieczka, G., Nachman, B. & Plehn, T. GANplifying event samples. *SciPost Phys*. **10**, 139. 10.21468/SciPostPhys.10.6.139 (2021).

[25] Otten S (2021). Event generation and statistical sampling for physics with deep generative models and a density information buffer. Nat. Commun..

[26] Carrazza S, Dreyer FA (2019). Lund jet images from generative and cycle-consistent adversarial networks. Eur. Phys. J. C.

[27] Di Sipio R, Faucci Giannelli M, Ketabchi Haghighat S, Palazzo S (2019). DijetGAN: A generative-adversarial network approach for the simulation of QCD Dijet events at the LHC. JHEP.

[28] Paganini M, de Oliveira L, Nachman B (2018). CaloGAN: Simulating 3D high energy particle showers in multilayer electromagnetic calorimeters with generative adversarial networks. Phys. Rev. D..

[29] Gao C, Höche S, Isaacson J, Krause C, Schulz H (2020). Event generation with normalizing flows. Phys. Rev. D..

[30] Krause, C. & Shih, D. CaloFlow: Fast and accurate generation of calorimeter showers with normalizing flows. arXiv e-prints. 10.48550/arXiv.2106.05285 (2021).

[31] Krause, C. & Shih, D. CaloFlow II: Even faster and still accurate generation of calorimeter showers with normalizing flows. arXiv e-prints. 10.48550/arXiv.2110.11377 (2021).

[32] Cheng T, Arguin J-F, Leissner-Martin J, Pilette J, Golling T (2023). Variational autoencoders for anomalous jet tagging. Phys. Rev. D.

[33] Deep generative models for fast shower simulation in ATLAS. Tech. Rep., CERN, Geneva (2018). All figures including auxiliary figures are available at https://atlas.web.cern.ch/Atlas/GROUPS/PHYSICS/PUBNOTES/ATL-SOFT-PUB-2018-001.

[34] Liu J, Qi Y, Meng ZY, Fu L (2017). Self-learning Monte Carlo method. Phys. Rev. B.

[35] Huang L, Wang L (2017). Accelerated Monte Carlo simulations with restricted Boltzmann machines. Phys. Rev. B.

[36] Shen H, Liu J, Fu L (2018). Self-learning Monte Carlo with deep neural networks. Phys. Rev. B.

[37] Wu D, Rossi R, Carleo G (2021). Unbiased Monte Carlo cluster updates with autoregressive neural networks. Phys. Rev. Res..

[38] Stratis, G., Weinberg, P., Imbiriba, T., Closas, P. & Feiguin, A. E. Sample generation for the spin-fermion model using neural networks. arXiv e-prints (2022). eprint2206.07753.

[39] Selvaggi M (2014). DELPHES 3: A modular framework for fast-simulation of generic collider experiments. J. Phys. Conf. Ser..

[40] Grazzini M, Kallweit S, Wiesemann M (2018). Fully differential NNLO computations with MATRIX. Eur. Phys. J. C.

[41] Gehrmann T, von Manteuffel A, Tancredi L (2015). The two-loop helicity amplitudes for $$q{{\overline{q}}^{\prime}} \to V_{1} V_{2} \to 4$$ leptons. JHEP.

[42] Maas, A. L. Rectifier nonlinearities improve neural network acoustic models (2013).

[43] Nair, V. & Hinton, G. E. Rectified linear units improve restricted boltzmann machines. ICML’10, 807-814 (Omnipress, Madison, WI, USA, 2010).

[44] Sun, Y., Wang, X. & Tang, X. Deeply learned face representations are sparse, selective, and robust. CoRR e-prints (2014). eprint1412.1265.

[45] Clevert, D., Unterthiner, T. & Hochreiter, S. Fast and accurate deep network learning by exponential linear units (elus). in *4th International Conference on Learning Representations, ICLR 2016, San Juan, Puerto Rico, May 2-4, 2016, Conference Track Proceedings* (2016). arXiv:1511.07289.

[46] Kingma, D. P. & Ba, J. Adam: A method for stochastic optimization. in *3rd International Conference on Learning Representations*, ICLR 2015, San Diego, CA, USA, May 7-9, 2015, Conference Track Proceedings (2015). arXiv:1412.6980.

[47] Glorot, X. & Bengio, Y. Understanding the difficulty of training deep feedforward neural networks. in (Teh, Y. W. & Titterington, M. eds.) *Proceedings of the Thirteenth International Conference on Artificial Intelligence and Statistics*, vol. 9 of Proceedings of Machine Learning Research, 249–256 (PMLR, Chia Laguna Resort, Sardinia, Italy, 2010).

[48] Srivastava, R. K., Greff, K. & Schmidhuber, J. Highway networks. CoRR e-prints (2015). eprint1505.00387.

[49] He, K., Zhang, X., Ren, S. & Sun, J. Deep residual learning for image recognition. in *2016 IEEE Conference on Computer Vision and Pattern Recognition (CVPR)*, 770–778. 10.1109/CVPR.2016.90 (2016).

[50] Zagoruyko, S. & Komodakis, N. Wide residual networks. in (Richard C. Wilson, E. R. H. & Smith, W. A. P. eds.) *Proceedings of the British Machine Vision Conference (BMVC)*, 87.1–87.12. 10.5244/C.30.87 (BMVA Press, 2016).

[51] Huang, G., Liu, Z., Van Der Maaten, L. & Weinberger, K. Q. Densely connected convolutional networks. in *2017 IEEE Conference on Computer Vision and Pattern Recognition (CVPR)*, 2261–2269. 10.1109/CVPR.2017.243 (2017).

[52] Xie, S., Girshick, R., Dollár, P., Tu, Z. & He, K. Aggregated residual transformations for deep neural networks. in *2017 IEEE Conference on Computer Vision and Pattern Recognition (CVPR)*, 5987–5995. 10.1109/CVPR.2017.634 (2017).

[53] Chen, T. & Guestrin, C. XGBoost: A scalable tree boosting system. in *Proceedings of the 22nd ACM SIGKDD International Conference on Knowledge Discovery and Data Mining, KDD ’16*, 785–794. 10.1145/2939672.2939785 (ACM, New York, NY, USA, 2016).

[54] Abadi, M. et al. TensorFlow: Large-scale machine learning on heterogeneous systems (2015). Software available from tensorflow.org.

[55] Aghli, N. & Ribeiro, E. Combining weight pruning and knowledge distillation for CNN compression. in *2021 IEEE/CVF Conference on Computer Vision and Pattern Recognition Workshops (CVPRW)*, 3185–3192. 10.1109/CVPRW53098.2021.00356 (2021).

[56] Cheng Y, Wang D, Zhou P, Zhang T (2018). Model compression and acceleration for deep neural networks: The principles, progress, and challenges. IEEE Signal Process. Mag..

[57] ATLAS HL-LHC Computing Conceptual Design Report. Tech. Rep. CERN-LHCC-2020-015, LHCC-G-178, CERN, Geneva (2020).

[58] ATLAS Software and Computing HL-LHC Roadmap. Tech. Rep. CERN-LHCC-2022-005, LHCC-G-182, CERN, Geneva (2022).

